# Mesoporous Silicas with Tunable Morphology for the Immobilization of Laccase

**DOI:** 10.3390/molecules19067057

**Published:** 2014-05-30

**Authors:** Victoria Gascón, Isabel Díaz, Carlos Márquez-Álvarez, Rosa M. Blanco

**Affiliations:** Instituto de Catálisis y Petroleoquímica, CSIC, Marie Curie 2, Madrid 28049, Spain

**Keywords:** amorphous silica, biocatalysts, enzyme immobilization, functionalization, grafting, laccase, MS-3030, ordered mesoporous materials, SBA-15, support

## Abstract

Siliceous ordered mesoporous materials (OMM) are gaining interest as supports for enzyme immobilization due to their uniform pore size, large surface area, tunable pore network and the introduction of organic components to mesoporous structure. We used SBA-15 type silica materials, which exhibit a regular 2D hexagonal packing of cylindrical mesopores of uniform size, for non-covalent immobilization of laccase. Synthesis conditions were adjusted in order to obtain supports with different particle shape, where those with shorter channels had higher loading capacity. Despite the similar isoelectric points of silica and laccase and the close match between the size of laccase and the pore dimensions of these SBA-15 materials, immobilization was achieved with very low leaching. Surface modification of macro-/mesoporous amorphous silica by grafting of amine moieties was proved to significantly increase the isoelectric point of this support and improve the immobilization yield.

## 1. Introduction

Siliceous ordered mesoporous materials (OMMs) are gaining interest as supports for enzyme immobilization because of their uniform and tunable pore size and large surface area, which make them excellent hosts for the adsorption of bulky molecules like enzymes [1,2,3,4,5]. OMMs are thermally, mechanically and chemically stable and insoluble in the solution used for the immobilization process and their surface can be grafted with different functionalities [6,7,8,9,10]. The immobilization of enzymes in functionalized OMMs can increase the operational stability, durability and importantly, the catalyst particles can be easily separated and reused without loss of catalytic activity [11,12].

To evaluate the suitability of a mesoporous material for enzyme immobilization the following factors are to be considered: pore diameter, surface area, pore volume, particle size, morphology, as well as surface modification. The effects of pore size and enzyme-support material interactions have demonstrated to have significant roles in the enzyme loading and activity. Surface chemical modification of the support could further enhance interactions between the pore walls and the enzyme [12]. In addition, the diffusion phenomena related to particle size are greatly affected by the support morphology [4,13].

Enzymes can be immobilized in mesoporous materials by different methods: adsorption, covalent attachment or encapsulation [4]. Covalent attachment is not advisable when pore size is close to enzyme dimension because of a “stopper” effect which may result in low immobilization yield, and always requires chemical modification of the enzyme which may result in catalytic activity decay. Adsorption seems more suitable because both drawbacks are avoided. However the driving forces of non-covalent immobilization are relatively weak interactions and, therefore, a careful design of the mesoporous support is necessary. 

Laccases (E.C. 1.10.3.2) are a group of multicopper enzymes of industrial interest that catalyze the oxidation of phenolic compounds such as *ortho*- and *para*-diphenols to their corresponding quinones with the concomitant reduction of oxygen to water [14,15,16,17,18]. Their potential as biocatalysts for use in several biotechnological processes, in nanobiotechnology applications, in food processing and in green chemistry [19,20,21,22,23,24,25,26] is limited by their low operational stability, mostly due to their rapid inactivation by different factors [12]. 

In this article we deal with the synthesis of SBA-15 type mesoporous silica supports with different particle morphology offering improved diffusional properties. SBA-15 with conventional rod type of particles, showing long channels (SBA-15-L) is compared with hexagonal particles formed by short mesochannels (SBA-15-S). Finally, we evaluate the incorporation of amine groups in large pore-size amorphous silica via grafting method (MS-3030-N). The morphology of SBA-15 structure, the effect of aminopropyl surface groups on the laccase immobilization behavior and detailed characterization results will be discussed in order to shed some light on the immobilization of enzymes. The laccase immobilization yields on pure silicas and the functionalized amorphous silica, specific activity measurements as well as enzyme leaching tests of immobilized laccase are also reported.

## 2. Results and Discussion

### 2.1. Characterization of Supports

The SBA-15 materials were characterized by X-ray diffraction, scanning and transmission electron microscopy to evaluate the morphology and internal structure of the particles, and nitrogen adsorption, to obtain the textural properties of the supports.

**Figure 1:**
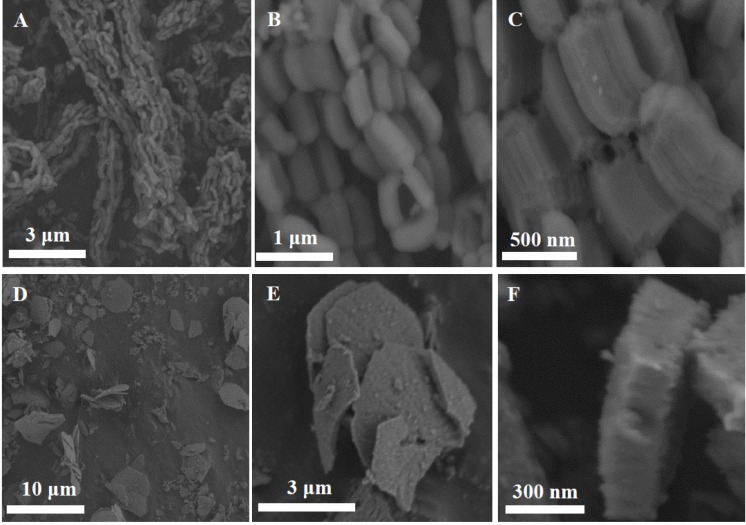
SEM micrographs of mesoporous silicas: SBA-15-L (**A**–**C**), SBA-15-S (**D**–**F**).

X-ray diffraction patterns of the SBA-15 materials synthesized with different surfactants, conditions and silica sources corroborated the 2D *p*6*mm* mesostructure with a unit cell parameter *a_o_* of 11.1 nm (data not shown). The desired effect of morphology modification was observed by scanning electron microscopy. SEM images of SBA-15-L and SBA-15-S are shown in Figure 1. The structure of SBA-15-L particles is fiber-like or elongated rod, formed by aggregates of rods connected into ropelike macrostructures (Figure 1A). The average particle size is 0.75–1 µm in length and about 2 µm in width (Figure 1B,C). SBA-15-S particles exhibit hexagonal dish-shaped morphology or short rod (Figure 1D–F). The average particle size is 5 µm in diameter and 0.2 µm width. Moreover, the alignment of channels with the axial direction of the rod-like particles on SBA-15-L and SBA-15-S could be corroborated by transmission electron microscopy (TEM). 

**Figure 2:**
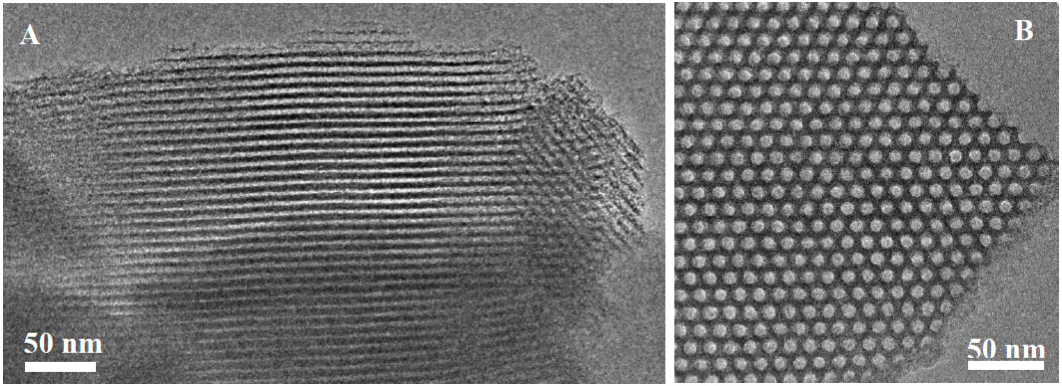
TEM images of calcined SBA-15-L in the direction perpendicular to the pore axis (**A**) and SBA-15-S in the direction of the pore axis (**B**).

Figure 2 displays representative TEM images of these materials showing the projection in the direction perpendicular to the channels in the longitudinal axis of sample SBA-15-L (Figure 2A). The hexagonal symmetry of the pores, like a honeycomb, characteristic of this kind of symmetry is also observed when the image is taken in the direction parallel to the mesochannels and along the short axis of the dish in sample SBA-15-S (Figure 2B). In both cases, the images allow observing the respective morphology, long rods in SBA-15-L and hexagonal dishes in SBA-15-S.

**Figure 3:**
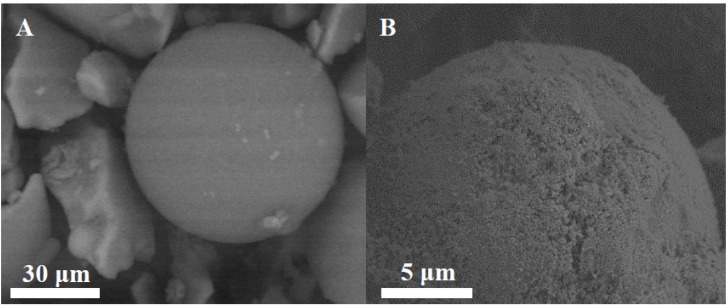
SEM micrographs of amorphous silicas: MS-3030 (**A**) and MS-3030-G-N (**B**).

XRD patterns of commercial amorphous silica, MS-3030, and amino-functionalized sample, MS-3030-N, show disordered structure with no reflections at low diffraction angles (data not shown). These materials were used to compare the effect of the structure and pore diameter with OMMs. Figure 3 shows the SEM images of MS-3030 (Figure 3A) and MS-3030-N (Figure 3B) revealing fragmented spherical particles with diameter around 74 µm, indicating that there is no alteration in silica morphology after amine-functionalization with aminopropyltriethoxysilane (APTS).

**Figure 4:**
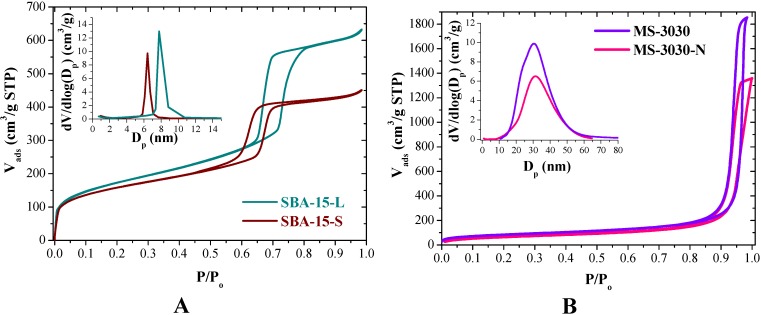
N_2_ adsorption-desorption isotherms and pore size distributions: (**A**) SBA-15-L and SBA-15-S; (**B**) MS-3030 and MS-3030-N.

SBA-15-L and SBA-15-S exhibit type IV nitrogen adsorption-desorption isotherms with hysteresis loops of H1 type associated with capillarity condensation at high relative pressure (P/P_o_ 0.7–0.8), which is characteristic of mesoporous materials with hexagonal arrangement of cylindrical pore channels (Figure 4A). Pore size distribution curves calculated from the adsorption branch of the isotherms are shown in the inset of Figure 4A. The curves show very narrow pore size distribution with 7.8 and 6.4 nm mean pore size, respectively. SBA-15-L has a surface are of 609 m^2^/g and pore volume of 1.0 cm^3^/g and SBA-15-S has 550 m^2^/g and 0.7 cm^3^/g, respectively. 

The N_2_ isotherms of amorphous silica before and after functionalization (MS-3030 and MS-3030-N) are shown in Figure 4B. Both materials exhibited type IV isotherms, corresponding to mesoporous materials. The pore size distribution curves of amorphous materials show a wider range, indicating heterogeneity in the porous structure with average pore diameter of 30 nm. As compared to MS-3030, MS-3030-N exhibited an expected slight decrease in BET surface area (from 296 to 236 m^2^/g) and pore volume (from 2.5 to 2.1 cm^3^/g) due to grafting of aminopropyl organic chains. The incorporation of the amine functional groups by grafting has been confirmed by elemental analysis yielding a 1.81 mmol N per gram of SiO_2_ in MS-3030-N material.

### 2.2. Immobilization of Laccase and Specific Activity

**Figure 5:**
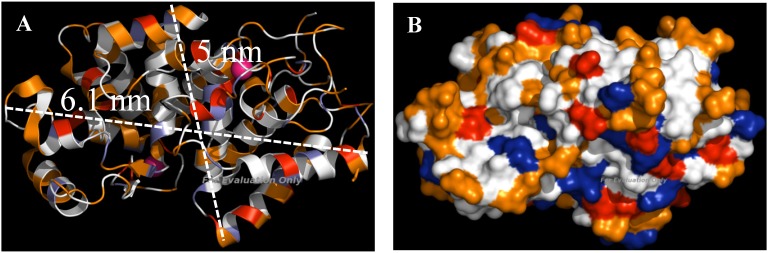
(**A**) Average dimensions of laccase measured in PyMOL (~6.1 nm × 5.0 nm × 4.9 nm). (**B**) Surface distribution of amino acids in laccase constructed in PyMOL (blue, basic amino acids; red, acidic amino acids; orange, polar amino acids; white, nonpolar amino acids; pink, copper atoms).

The laccase used in this work was manufactured by pure culture fermentation of a genetically modified strain of *Aspergillus oryzae* that contains the laccase gene derived from *Myceliophthora thermophila*. Bioinformatics analysis was used to determine the aminoacid sequence and 3D structure of this commercial laccase because the crystal structure is not solved yet and its dimensions have not been reported. An image of the laccase has been reconstructed and visualized using the software package PyMOL [27]. With the use of PyMOL and repeated rotations of the enzyme molecule, the average molecular size of laccase was found to be approximately 6.1 nm × 5.0 nm × 4.9 nm (Figure 5A). 

The molecular weight estimates for the laccase obtained from SDS-PAGE gel electrophoresis is around 80 kDa, consistent with other authors [28]. According to the enzyme dimensions and to the modelling studies and the textural properties of the supports, adsorption inside the pores of SBA-15 materials should be enabled. The surface distribution of amino acids in laccase is displayed in Figure 5B.

Electrostatic interactions between laccase molecules and the inner mesopore wall were considered for enzyme immobilization. Immobilization of laccase was achieved by suspending purely siliceous materials (SBA-15-L, SBA-15-S and MS-3030) in laccase solution at pH 3.5. In these conditions, laccase molecules bear a positive net charge (isoelectric point (pI) of laccase is 4.2). The silanol groups inside the mesopores are deprotonated at pH 3.5 bearing a negatively charged surface, because their isoelectric point is about 2.0. 

**Figure 6:**
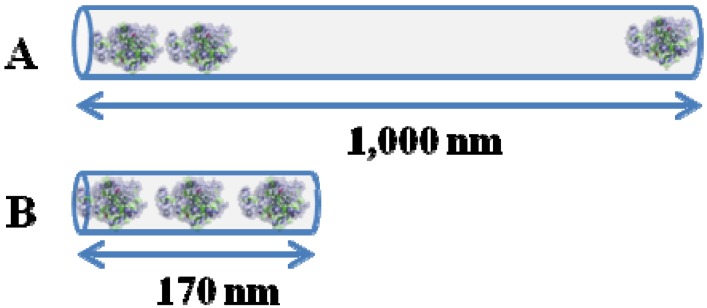
Schematic illustration of the laccase immobilization on SBA-15-L mesochannels (**A**) and SBA-15-S mesochannels (**B**).

Both ordered materials are similar in textural properties. However, despite the moderately lower surface area and pore diameter of SBA-15-S, this support exhibited moderately higher adsorption capacity compared to SBA-15-L (38.41 mg/g *vs.* 31.28 mg/g). The main difference comes from the different support morphology highlighting the influence of this parameter on laccase immobilization. A schematic illustration is proposed (Figure 6) showing the immobilization of laccase on the two different supports. Laccase dimensions are of similar magnitude than channels pore size, so the presence of an enzyme molecule inside may prevent the access of another one, acting as a stopper or at least making enzyme diffusion more difficult. Therefore, despite the smaller pore diameter of SBA-15-S, its shorter channels (of about 170 nm) can accommodate more enzyme molecules per unit length than the longer ones of SBA-15-L (1,000 nm length) where a part of the inner surface of the channel is not accessible to laccase molecules. This also explains why this material having a higher surface area does not result in a higher immobilization yield. This behavior has also been observed by other authors. According to Fan and coworkers [29], a SBA-15 sample with rod-like macromorphology displays faster loading kinetics and a higher loading capacity for lysozyme than an SBA-15 sample with fiber-like morphology. So, the morphology of OMM particles plays a significant role in the efficiency of enzyme immobilization.

Regarding specific activity, the longer channels seem to challenge the diffusion of substrates and products more than the shorter ones, as suggested by the lower value in the long-channel SBA-15-L.

**Figure 7:**
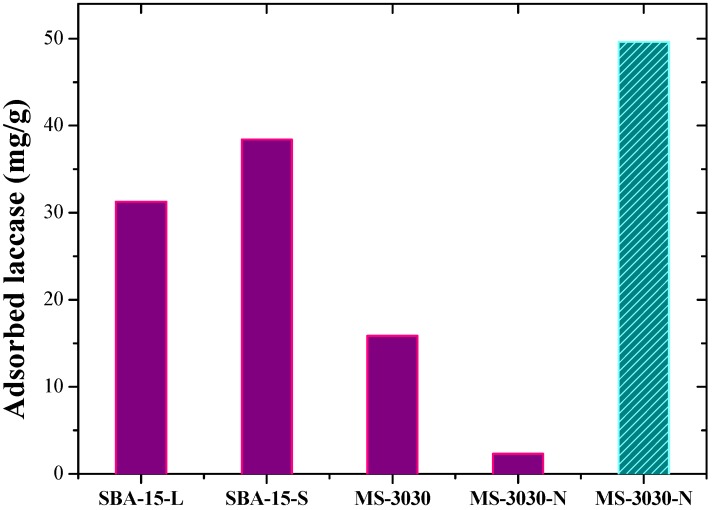
Adsorption capacity of different supports. Solid bars: immobilization at pH 3.5. Dashed bar: immobilization at pH 5.5.

The isoelectric points of the support and the enzyme are close, so the charge densities at pH 3.5 are low. Grafting the support with amine groups shifts its pI to higher value, so the interval becomes larger Protonated terminal amine groups can interact with deprotonated residues of aspartic acid and glutamic acid (pKa 4) at pH 5.5 which is an intermediate value between isoelectric points of enzyme and amino-modified support, and it is far from these values. Therefore the respective densities of charge should be higher than in the systems enzyme-siliceous supports at pH 3.5. These high charge densities should drive stronger electrostatic interactions and favor the immobilization of laccase. However, the close sizes of enzyme and pore diameter does not enable to functionalize SBA-15 materials while leaving enough room for enzyme diffusion, therefore a commercial siliceous material, MS-3030, with wider pores was used for comparison. The 30 nm average pore size of this amorphous material permits functionalization without compromising the access or diffusion of enzyme molecules. Figure 7 and Table 1 displays the adsorption of laccase on purely siliceous materials (SBA-15-L, SBA-15-S and MS-3030 at pH 3.5) and amine grafted amorphous silica (MS-3030-N at pH 3.5 and pH 5.5) for initial amount of enzyme solution of 50 mg/g support.

**Table 1:** Laccase immobilization characteristics and catalytic results for initial amount of enzyme solution of 50 mg/g support.

Support	pH immob. ^a^	*t_c_* (h) ^b^	% Immob. ^c^	Enzyme loading (mg/g) ^d^	Specific activity (U/mg) ^e^
**SBA-15-L**	3.5	3	62.56	31.28	0.134
**SBA-15-S**	3.5	3	76.82	38.41	0.159
**MS-3030**	3.5	3	31.76	15.88	0.014
**MS-3030-N**	5.5	3	99.28	49.64	0.981

^a^ pH of immobilization; ^b^ The time contact (*t_c_*) is the time required to reach a constant activity of the supernatant towards the oxidation of syringaldazine, indicative of maximum loading in the solid; ^c^ percent yield of immobilization; ^d^ The enzyme loading is expressed in milligrams of enzyme per gram of material; ^e^ The specific activity is expressed in units of syringaldazine converted per milligram of enzyme inside the material. Specific activity of soluble laccase: 5.68 U/mg.

Laccase was also adsorbed on purely siliceous MS-3030 at pH 3.5 with poor results: 15.88 mg/g in the same immobilization time of 3 h. The pore tortuosity and large particle size of this material may result in a slower diffusion of enzyme through the pore network despite the wider average pore size of 30 nm. Immobilization on MS-3030-N at pH 3.5 was negligible (2.32 mg/g, results not shown) because of electrostatic repulsion between the positively charged enzyme and the protonated amino groups of the support. However, immobilization on MS-3030-N performed at pH 5.5 gave a 99% yield despite the surface area is only 296 m^2^/g. At this pH, enzyme and support surfaces have opposite charges and high charge densities, which favors strong electrostatic interactions. This phenomenon indicates that amino groups are appropriate for laccase immobilization at a suitable pH. 

The activity of the biocatalysts was tested in the oxidation of syringaldazine. Table 1 shows that the presence of the amine moieties in MS-3030-N also improves the specific activity of the laccase. SBA-15-L and SBA-15-S display higher activity than MS-3030, probably due to slower diffusion of substrate and product through the tortuous porous network and the larger particle size of this latter material. In contrast, SBA-15 materials offer an ordered structure with high pore connectivity and they are assembled with smaller particles than amorphous silica, which should favor a shorter diffusion course [30]. Other authors have also shown that materials with pore sizes much larger than the enzyme size have an adverse effect on enzyme activity [31].

### 2.3. Leaching Test of Immobilized Laccase

**Figure 8:**
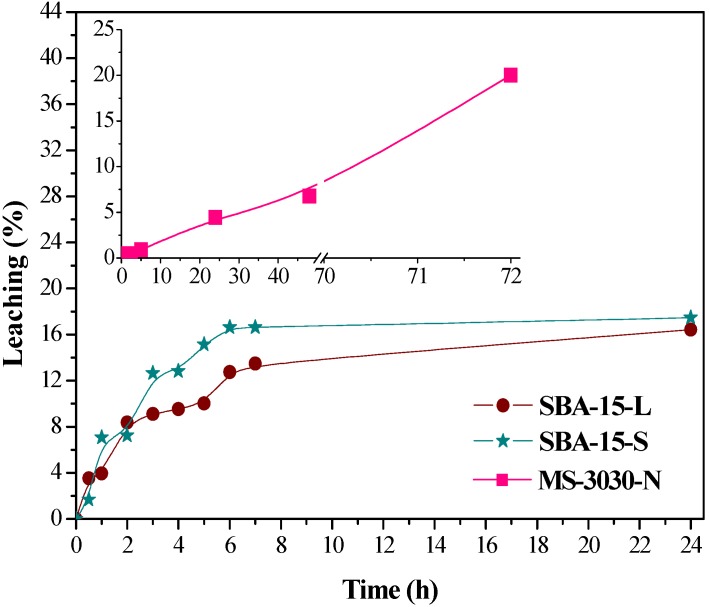
Leaching of enzyme from materials, expressed as percent of the initial enzyme loading released as a function of time.

The extent of leaching of laccase from the inner structure of supports in aqueous media and the presence of laccase inside the porous network of the materials after the leaching tests have been studied due to the non-covalent nature of the enzyme-support binding through electrostatic interactions. Biocatalysts were incubated in aqueous solution of low ionic strength buffer and high dilution at pH 4.5. In these conditions, weak repulsion is established between negatively charged SBA-15-L or SBA-15-S and laccase, and weak attraction is still maintained between the positively charged MS-3030-N support and negatively charged laccase. In both cases low ionic strength buffered solution is used just to preserve pH without forcing enzyme leaching due to ionic competition anion-enzyme. These conditions are expected to favor enzyme desorption from the siliceous materials more than from the amino-modified one. Figure 8 summarizes these results, plotting the percentage of the initial enzyme loading leached with time. 

Two different kinds of patterns can be distinguished. Leaching profile in SBA-15-L and SBA-15-S showed fast initial desorption values and a plateau suggesting that the narrow pore size and confinement of the laccase minimize leaching of the enzyme from the inner surfaces. The first initial slope is probably due to enzyme molecules immobilized on external surfaces or close to the pore edges of the OMM particles. 

Despite the electrostatic attraction between the enzyme molecules and functional groups on the surface remaining at pH 4.5, amine amorphous silica (MS-3030-N) shows a continuous and fast release of enzyme, without reaching an equilibrium point in the range of time studied (up to 72 h). This material with much larger pore diameter offers no restriction to enzyme diffusion and is highly susceptible to leaching during the operation. The confinement of the enzyme inside pores seems to be more efficient to prevent leaching than the intensity of interaction with support. 

**Figure 9:**
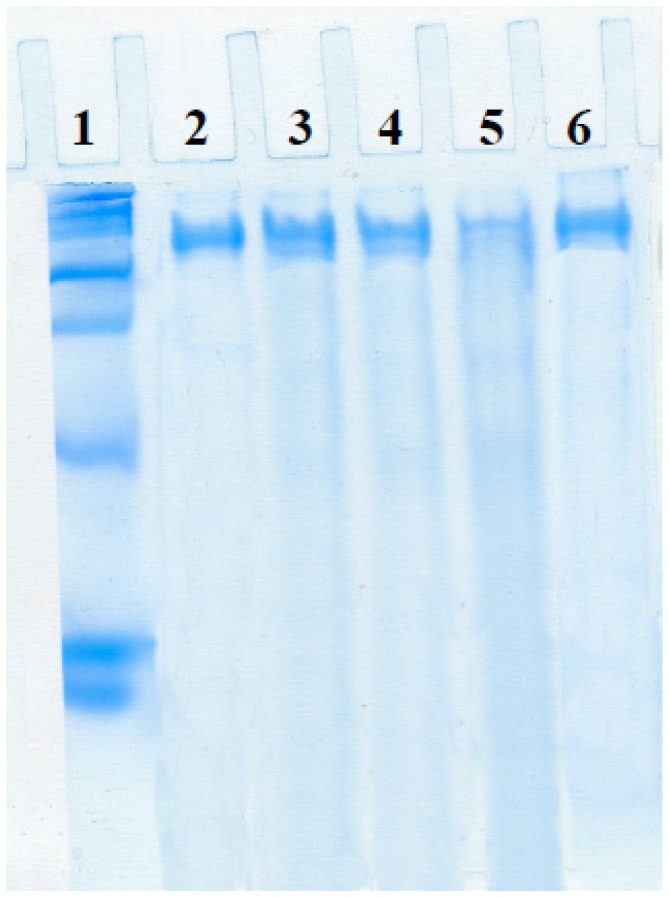
Electrophoresis in standard conditions. **1**: Protein standard (high range SDS-Page standard stained with coomassie G-250 stain; **2**: Soluble laccase; **3**: SBA-15-L; **4**: SBA-15-S; **5**: MS-3030; **6**: MS-3030-N.

From leaching tests, some laccase molecules seem to be adsorbed on the external surface of SBA-15 particles. In order to check the presence of enzyme inside particles, the biocatalysts previously incubated under the leaching conditions described above were treated in denaturing conditions of SDS-PAGE and their supernatants were assayed for electrophoresis. Linear polypeptide chain should easily exit the pores after this treatment and be detected in the supernatants. Bands were observed for all the samples which confirm that the enzyme is adsorbed inside the pores of amorphous silica and both SBA-15 materials (Figure 9). Leaching profile of MS-3030 is not displayed because laccase molecules were released from the support during biocatalyst washing due to wide pores and weak attraction; moreover the protein band obtained by electrophoresis is very thin (Figure 9, lane 5).

## 3. Experimental

### 3.1. Synthesis of the Supports

*Synthesis of SBA-15-L.* Pure silica SBA-15 with elongated rod particles was synthesized by using Pluronic P123 triblock copolymer according to the bibliography [32]. First, Pluronic P123 (4 g) was dissolved at room temperature in 1.9 M aqueous HCl solution (125 mL). The resulting solution was slowly stirred at 25 °C until the solution became clear. TEOS (9.25 mL) was then added to the solution and the resulting mixture was vigorously stirred at 40 °C for 20 h. Aging was then performed at 110 °C under static conditions for 24 h in a closed Teflon container. Finally, the white solid product was recovered by filtration, washed with water, air dried at room temperature and calcined at 550 °C in air for 5 h in a furnace for removal of the surfactant. 

*Synthesis of SBA-15-S*. Pure silica SBA-15 with dish-shaped particles was synthesized using Pluronic PE-10400 as structure directing agent and with tetramethylorthosilicate (TMOS) as the silica source. This material has prepared according to the method reported by Linton *et al.* [33,34]. PE-10400 (2.5 g) was dissolved in 1.6 M aqueous HCl solution (97.5 g). The mixture was stirred at room temperature in a closed Teflon container until a homogeneous clear solution was obtained. Then, the solution was heated at 55 °C for 1 h, after which TMOS (3.8 g) was added and stirred vigorously at 55 °C for 24 h. Subsequently, the container was transferred to an oven and kept at 80 °C for 24 h under static conditions. The resultant product was filtered, washed thoroughly with dry ethanol and air-dried at room temperature overnight. Finally, the surfactant template was removed by calcination at 550 °C for 5 h in air. 

*Functionalization of MS-3030 by grafting with aminopropyl groups* *(MS-3030-N)*. Commercial mesoporous amorphous silica material, MS-3030 manufactured by PQ Corporation (Conshohocken, PA, USA), was functionalized with aminopropyl groups by silanization reaction of surface silanol groups with aminopropyltriethoxysilane (APTES) [8,35]. Silica powder (5 g) was placed in a round bottom flask and degassed at 80 °C under vacuum for 20 h to remove adsorbed water [7]. This dried material was dispersed in dry toluene (100 mL), and APTES (25 g) was added under constant stirring. The reaction mixture was refluxed under stirring at atmospheric pressure in a nitrogen atmosphere for 24 h. After a slow cooling of the reaction mixture, the resulting amino-functionalized material was recovered by filtration, washed twice with dry toluene for removal of unreacted reagent, followed by three times with acetone, and finally dried under vacuum for 24 h. 

### 3.2. Characterization Techniques

X-ray diffraction (XRD) patterns were acquired with a X’PERT diffractometer (PANalytical, Almelo, The Netherlands) using the Cu-K_α_ (λ = 1.5406 Å) radiation. The data were registered with two theta step size 0.02° and accumulation time 20 s. Transmission electron micrographs (TEM) were obtained on a JEM-2100 electron microscope (JEOL, Akishima, Japan) operated at 200 kV. A small amount of sample powder was dispersed in ethanol and dropped on a holey carbon coated copper grid. Scanning electron microscopy (SEM) images were collected with a Nova NanoSEM 230 FE-SEM microscope (FEI, Eindhoven, The Netherlands) with vCD detector. The samples were prepared by placing material powder on double-sided graphite adhesive tape mounted on the sample holder. Nitrogen adsorption-desorption isotherms were measured at −196 °C on an ASAP 2420 device (Micromeritics, Norcross, GA, USA). All calcined silicas before enzyme immobilization were outgassed at 350 °C for 16 h under high vacuum prior to the measurements. The specific surface area, *S_BET_*, was calculated from nitrogen adsorption data in the relative pressure range from 0.04 to 0.2 using the BET (Brunauer-Emmett-Teller) method. The total pore volume, *V_p_*, was determined from the amount of nitrogen adsorbed at a relative pressure of 0.97. Pore size distributions were determined from the adsorption branch of the N_2_ isotherms using the BJH (Barrett-Joyner-Halenda) model with cylindrical geometry of the pores. The BJH pore diameter, D_p BJH_, is defined as the position of the maximum of the pore size distribution. Quantitative determination of the nitrogen content of organo-functionalized support was performed using a CHNS-932 Elemental Analyser (LECO Corporation, St. Joseph, MI, USA) with an AD-4 autobalance (Perkin Elmer, Shelton, CT, USA). Thermogravimetric analyses were carried out using a TGA 7 instrument (Perkin Elmer). Samples were heated in air atmosphere from 25 to 900 °C at a rate of 20 °C/min.

### 3.3. Protein Determination and Activity Assay

Protein content of commercial laccase extract was determined with the Bio-Rad Protein Assay (Bio-Rad, München, Germany), based on the Bradford assay [36], using bovine serum albumin (BSA) as protein standard. This extract was found to contain a protein concentration of 1.3 mg/mL. Electrophoresis (lane 2 in Figure 9) showed a unique band, so all protein was assigned to laccase.

Enzymatic activity of free and immobilized laccase was determined spectrophotometrically by measuring the increase in absorbance at 530 nm caused by the oxidation of syringaldazine in the presence of atmospheric oxygen to tetramethoxyazobis(methylene) quinone [37,38], using an 8453 UV-Vis spectrophotometer (Agilent Technologies, Waldbronn, Germany) equipped with a stirring device and temperature control. The reaction mixture consisted of 2200 µL of potassium dihydrogen phosphate/disodium hydrogen phosphate buffer (pH 6.5, 100 mM), 60 µL of 1 mM syringaldazine solution dissolved in ethanol. 100 µL of enzyme solution, suspension or supernatant, were added to the cuvette containing the above substrate solution under stirring and the reaction was monitored continuously for 5 min by measuring the absorption at 530 nm at 30 °C. One laccase activity unit (U) is defined as the amount of enzyme required to oxidize 1 µmol of syringaldazine per minute at 30 °C (ε_530_ nm = 65,000 M^−1^ cm^−1^). Each sample was measured three times, and the average value was used.

### 3.4. Immobilization of Laccase on Mesoporous Silicates

Immobilization of laccase on purely siliceous supports, namely SBA-15-L, SBA-15-S and MS-3030, was performed by suspending the materials in enzyme solutions at pH 3.5 (50 mM citric acid/trisodium citrate buffer). Amino-functionalized material, MS-3030-N, was suspended in laccase solutions in 50 mM acetic acid/sodium acetate buffer at pH 5.5.

To 10 mL enzyme solutions (containing 2.5 mg laccase), the support (50 mg) was added and left in suspension under slow rotation at room temperature to allow immobilization to occur by adsorption. Aliquots were withdrawn at given times, and the enzymatic activity of the suspension and supernatant were analyzed by the syringaldazine oxidation assay. The decrease in activity of the supernatant to a minimum and constant value indicated the end point of the immobilization process. At this equilibrium point, the solid was filtered off and washed with 50 mM buffer (at the same pH as used for immobilization, pH 3.5 or 5.5). The solid samples were first dried under vacuum and afterwards under dry nitrogen stream, and then stored at 4 °C in the refrigerator for later analysis. A mass balance was applied to calculate the amount of enzyme immobilized on the supports.

To evaluate the enzymatic activity of laccase immobilized on mesoporous silicas, 10 mg of the respective dried biocatalysts were resuspended in 1 mL of 50 mM buffer (pH 3.5 for biocatalysts prepared with purely siliceous supports or pH 5.5 for those on amino-silica supports) and analyzed for remaining laccase activity by the syringaldazine oxidation assay. 

### 3.5. Immobilized Laccase Leaching Tests

To measure the leaching of laccase from the supports, samples of the solid containing the enzyme were suspended in 50 mM acetic acid/sodium acetate at pH 4.5 and the solid/liquid ratio was 1.25 mg/mL. At different times aliquots of the suspensions were withdrawn and centrifuged, and the protein content of the supernatants was measured according to Bradford assay [36].

### 3.6. SDS-PAGE Electrophoresis

An attempt to verify the location of laccase inside the porous network of the materials was made. Since solid samples are not suitable for electrophoresis, the enzyme was forced to exit the pores. First, the laccase immobilized on different materials was suspended in buffered solutions for 24 h at pH 4.5 as described above to desorb all protein molecules adsorbed on the external surface of support particles. The filtered solids were suspended in electrophoresis sample buffer (containing sodium dodecyl sulfate, mercaptoethanol, bromophenol blue, Tris/HCl buffer pH 6.8 and glycerol) in the same amounts as in electrophoresis samples, and boiled for 5 min. In such denaturing conditions, the tertiary structure of the protein should be lost and the lineal peptide chain should then be easily released from the pores. The supernatants of these suspensions after centrifuging were withdrawn and analyzed by SDS-PAGE electrophoresis [39].

## 4. Conclusions

Two similar ordered mesoporous materials with well-defined morphologies and highly uniform particle sizes synthesized using surfactant templates in acidic solutions were tested as supports for laccase immobilization. The morphology of SBA-15 demonstrated to play a significant role in the efficiency of enzyme immobilization. Short SBA-15-S channels proved to be more efficient to carry higher enzyme loading whereas restriction to enzyme diffusion in the longer SBA-15-L channels resulted in lower enzyme loading and higher retention (or less leaching). Also, some restriction to the diffusion of substrate or product may drive a lower specific activity. 

The relevance of the pore and particle features was also demonstrated by the comparison with an amorphous meso-macroporous siliceous material, where electrostatic interactions were strengthen by the presence of amine groups. Despite immobilization yield was higher in this material, leaching of the enzyme had no restriction. However, the confinement in SBA-15 channels was more efficient to retain enzyme.

Our results on the immobilization of laccase on ordered mesoporous materials (SBA-15-L and SBA-15-S) demonstrate the importance of matching pore diameter and enzyme dimensions and chemical affinity (MS-3030-N). Efficient immobilization of laccase can be improved when a detailed understanding of the enzyme properties is combined with a tailored design of mesoporous supports. The aim should be now to synthesize ordered mesoporous materials with larger pore diameter and to incorporate amine groups.
